# Gingerol, a Natural Antioxidant, Attenuates Hyperglycemia and Downstream Complications

**DOI:** 10.3390/metabo12121274

**Published:** 2022-12-16

**Authors:** Khalid Saad Alharbi, Muhammad Shahid Nadeem, Obaid Afzal, Sami I. Alzarea, Abdulmalik S. A. Altamimi, Waleed Hassan Almalki, Bismillah Mubeen, Saima Iftikhar, Luqman Shah, Imran Kazmi

**Affiliations:** 1 Department of Pharmacology, College of Pharmacy, Jouf University, Sakaka 72341, Saudi Arabia; 2 Department of Biochemistry, Faculty of Science, King Abdulaziz University, Jeddah 21589, Saudi Arabia; 3 Department of Pharmaceutical Chemistry, College of Pharmacy, Prince Sattam Bin Abdulaziz University, Al-Kharj 11942, Saudi Arabia; 4 Department of Pharmacology, College of Pharmacy, Umm Al-Qura University, Makkah 21955, Saudi Arabia; 5 Institute of Molecular Biology and Biotechnology (IMBB), The University of Lahore, Lahore 54000, Pakistan; 6 School of Biological Sciences, University of Punjab, Lahore 54000, Pakistan; 7 Department of Biochemistry, Faculty of Science, Hazara University, Mansehra 21300, Pakistan

**Keywords:** Hyperglycemia, *Zingiber officinale*, gingerol, 6-gingerol, nephropathy, neuropathy, retinopathy, periodontitis, cataract

## Abstract

Hyperglycemia is seen in approximately 68 percent of patients admitted to a medical intensive care unit (ICU). In many acute circumstances, such as myocardial infarction, brain, injury and stroke, it is an independent predictor of mortality. Hyperglycemia is induced by a mix of genetic, environmental, and immunologic variables in people with type 1 diabetes. These factors cause pancreatic beta cell death and insulin insufficiency. Insulin resistance and irregular insulin production cause hyperglycemia in type 2 diabetes patients. Hyperglycemia activates a number of complicated interconnected metabolic processes. Hyperglycemia is a major contributor to the onset and progression of diabetes’ secondary complications such as neuropathy, nephropathy, retinopathy, cataracts, periodontitis, and bone and joint issues. Studies on the health benefits of ginger and its constituent’s impact on hyperglycemia and related disorders have been conducted and gingerol proved to be a potential pharmaceutically active constituent of ginger (*Zingiber officinale*) that has been shown to lower blood sugar levels, because it possesses antioxidant properties and it functions as an antioxidant in the complicated biochemical process that causes hyperglycemia to be activated. Gingerol not only helps in treating hyperglycemia but also shows effectivity against diseases related to it, such as cardiopathy, kidney failure, vision impairments, bone and joint problems, and teeth and gum infections. Moreover, fresh ginger has various gingerol analogues, with 6-gingerol being the most abundant. However, it is necessary to investigate the efficacy of its other analogues against hyperglycemia and associated disorders at various concentrations in order to determine the appropriate dose for treating these conditions.

## 1. Introduction

Gingerol is the most active and abundant component of ginger. The therapeutic effects of ginger are attributed to a mixture of gingerol derivatives known as 6-gingerol, 8-gingerol, and 10-gingerol, which are responsible for ginger’s moderate pungency [1,2,3]. Ginger’s main pharmacologically active component is 6-gingerol [2,4]. The ginger plant is a member of the Zingiberaceae family. It is a Southeast Asian spice and condiment that is now used in many countries to flavor food [5,6]. The phytochemistry of ginger is what gives it its health-promoting qualities [7,8]. Fresh ginger was divided into two groups, volatiles and nonvolatiles, by Jolad et al. Sesquiterpene and monoterpenoid hydrocarbons are among the volatiles that give ginger its characteristic scent and flavor. Gingerols, shogaols, paradols, and zingerone are examples of non-volatile pungent compounds [9,10,11].

In both adults and children, hyperglycemia is a regular occurrence in the intensive care unit, and it has been associated with poor outcomes [12]. It has been proven in perioperative studies, coronary care, and prospective randomized trials that aggressive treatment of hyperglycemia with insulin lowers morbidity and death. [13,14]. As a result, glycemic management is now frequently used in clinical practice and is included in several worldwide treatment guidelines [15]. However, the potential of an increase in dangerous hypoglycemia episodes as a result of this rise might be to blame for the lack of benefit, if not outright damage, shown in several recent studies [16]. Despite some doubts about the effectiveness of glycemic control, everyone agrees that hyperglycemia should be avoided because it is the source of many other fatal diseases [17].

Research has been done in this context to examine gingerol’s role as an anti-hyperglycemic and therapeutic agent against diseases caused by hyperglycemia. In a 2021 study by Almatroodi et al. [18], treatment with 6-gingerol significantly reduced the raised levels of oxidative stress in streptozotocin (STZ)-induced diabetic rats, and subsequently enhanced antioxidant levels. Furthermore, STZ-induced diabetic rats had significantly higher-than-normal levels of MDA in their kidneys. Treatment with 6-gingerol dramatically reduced this. Chronic hyperglycemia caused a considerable rise in malondialdehyde in the kidneys of rats, according to a prior study. Another study found that in hyperglycemic rats, ginger effectively reduced blood glucose, serum cholesterol, low density lipoproteins (LDLs), very low density lipoproteins (VLDLs) and triglycerides, while raising high density lipoproteins (HDLs) [19].

Previous research on ginger found that it has a renoprotective effect by regulating lipid peroxidation and maintaining histopathological alterations. In diabetic rats, ginger has a shielding effect against kidney impairment. Furthermore, after therapy with ginger, histological investigation revealed that diabetes-induced glomerular degenerative alterations were reduced. The effects of ginger powder on diabetic nephropathy have been assessed using antioxidant capacity and lipid peroxidation measurements. Ginger reduces lipid peroxidation, and reduces renal nephropathy, according to this study [20,21].

In short, gingerol is one of the most efficient compounds for treating hyperglycemia and the numerous catastrophic disorders that follow from it. It offers tremendous promise in the realms of human health care and treatment of illness. In this context, we wanted to assess the progress made in the use of gingerol as an anti-hyperglycemic molecule, as well as its influence on other disorders linked to it.

## 2. Discovery, Source, Biosynthesis, Properties and Action Mechanism of Gingerol

J. C. Thresh isolated gingerol from the ginger plant’s rhizome in 1879 [22]. In 1917, 6-gingerol was discovered and recognized as the most powerful derivative. The phenylpropanoid pathway is involved in the biosynthesis of gingerol [23,24].

**Figure 1:**
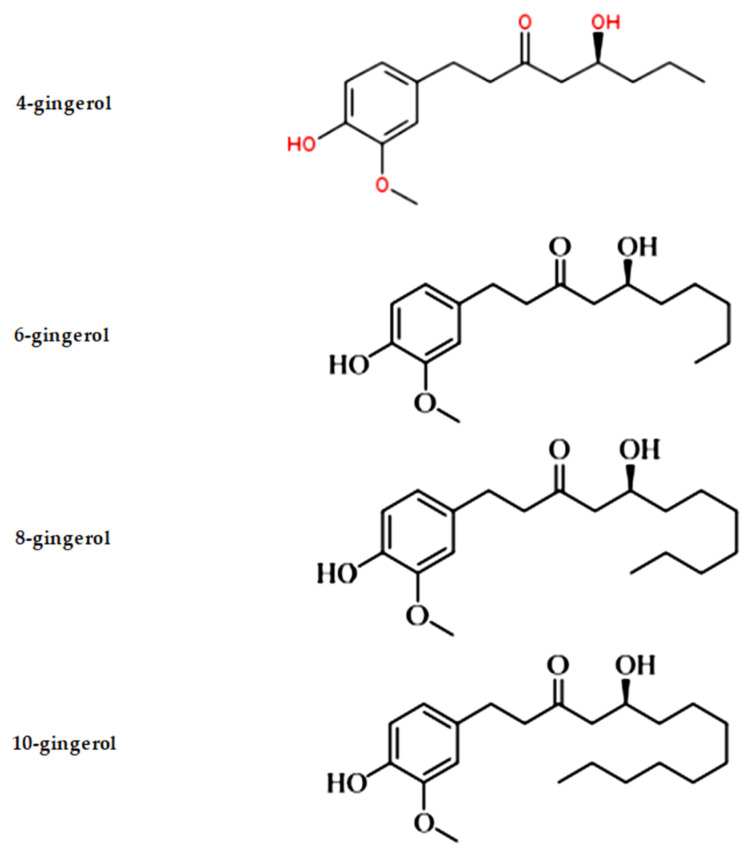
Structure of gingerols.

More than 70 analogues of gingerols have been discovered thus far [25]. Gingerol is a phenol phytochemical component found in fresh ginger that activates the tongue’s spice receptors. Cooking ginger converts gingerol to zingerone, which is less fragrant and has a spicy sweet scent, due to a reverse aldol process. Other varieties of gingerols, such as 8-gingerol, 10-gingerol, and 12-gingerol, are also found in ginger (Figure 1) [26,27]. Regarding gingerol’s metabolism, intravenous bolus investigations in rats showed that the serum protein binding of 6-gingerol was discovered to be greater than 90%, and the plasma concentration time curve of 6-gingerol was illustrated by a two-compartment open model. An intravenous bolus of 6-gingerol was quickly eliminated from the plasma o both healthy, normal rats and rats with acute renal failure. The terminal half-life ranged from 7.23 to 8.5 min. In rats with acute hepatic failure, the terminal phase of 6-gingerol rose noticeably to 11 min. Within 60 h, metabolites from a 50 mg/kg oral dosage of 6-gingerol were eliminated in the bile (48%) and urine (16%). It was discovered that a 100 mg/kg oral dose of Zingerone had elimination characteristics comparable to those of 6-gingerol, with 50% eliminated in the feces and 40% in the urine over the course of 24 h [28,29,30,31].

It is easy to convert 6-gingerol to (S)-[6]-gingerol-4′-O-glucuronide in the liver and intestinal epithelium after being administered orally to rats, and is then eliminated through the bile. Additionally, the urine contained the following six secondary metabolites: vanillic acid, ferulic acid, (S)-(+)-hydroxy-6-oxo-8-(4-hydroxy-3-methoxyphenyl), octanoic acid, 4-(4-hydroxy-3-methoxyphenyl) butanoic acid, and 9-hydroxy 6-gingerol [32].

### Hyperglycemia and Subsequent Complications

Hyperglycemia is derived from the Greek words hyper which means “high” and glykys which means “sweet/sugar” and haema which means “blood”. Hyperglycemia is defined as blood glucose levels of more than 125 mg/dL when fasting and more than 180 mg/dL 2 h after eating [33,34]. In the 17th century, Thomas Willis was the first to notice hyperglycemia during stressful situations [35].

Untreated hyperglycemia can lead to a variety of potentially life-threatening problems, including impairment to the eyes, kidneys, heart, vascular system, and nerves [36,37,38]. Hyperglycemia must be treated effectively and efficiently in order to reduce complications from sickness and improve patient outcomes [39]. Ketoacidosis (diabetic coma) can develop if hyperglycemia is not addressed. Ketoacidosis is a serious diabetes complication that occurs when the body produces too many ketones, which are blood acids [40,41].

## 3. Gingerol as an Antioxidant

As mentioned earlier, gingerol is known for its high potential antioxidative properties, and these properties are majorly responsible for the prevention and cure of a number of diseases. Masuda et al. 2004 found that gingerol has antioxidant and anti-inflammatory properties [42]. Ginger powder was found to contain 6-gingerol, 8-gingerol, 10-gingerol and 6-shogoals in quantities of 2.56 mg, 0.36 mg and 1.27 mg in 1 g, respectively [43]. The amount of 6-gingerol in ginger extract determines its antioxidant activity [44].

The capacity of the phenolic molecule to receive electrons and serve as a free radical scavenger by forming a stable phenoxyl radical may be connected to its antioxidant characteristics [45,46]. Patulin (PAT)-induced DNA strand breakage and micronuclei production were dramatically decreased by 6-gingerol in a study. PAT-induced intracellular ROS (reactive oxygen species) production and the level of 8-OHdG were also substantially decreased by 6-gingerol, which reduced the GSH depletion caused by PAT in HepG2 cells [47]. These results imply that 6-gingerol has a substantial protective potential against PAT-induced genotoxicity, and that its antioxidant activity may play a key role in reducing PAT-induced genotoxicity [47,48].

According to Kuhad et al. [49], 6-gingerol can protect rats against cisplatin-induced oxidative stress and renal impairment. It can act as a powerful antioxidant, improving renal function, decreasing lipid peroxidation, and boosting glutathione levels as well as superoxide dismutase and catalase activity. Furthermore, in the presence of iron (III) and ascorbate, 6-gingerol can reduce peroxidation of phospholipid liposomes [50]. Park et al. discovered that ROS are generated during the phenotypic change of fibroblasts into myofibroblasts, a process that promotes the development of nasal polyps by causing the buildup of extracellular matrix (ECM) [51]. In another study, sodium arsenite (iAs) was used to inhibit stress-induced insulin signaling in mice; 6-gingerol reduced high blood glucose levels and oxidative stress by increasing the levels of superoxide dismutase (SOD), catalase (CAT), glutathione peroxidase (GPx), and glutathione sulphate (GSH) [52]. As a result, 6-gingerol has the potential to become a significant natural antioxidant food additive [4].

## 4. Gingerol as a Curative Agent against Hyperglycemia

According to the International Diabetes Federation, there are currently 382 million individuals living with diabetes, and this number is expected to rise to 592 million by the end of 2035 [53] Diabetic nephropathy, a frequent late-stage consequence of diabetes mellitus, is associated with a high rate of morbidity and death. Furthermore, this disease is one of the most common microvascular impairments of diabetes mellitus [54], and it is characterized by persistent proteinuria, advanced loss of renal function, and morphological changes such as intestinal fibrosis, glomerular hypertrophy, glomerular membrane thickening, and mesangial expansion [54,55]. The significance of early blood glucose management in the prevention of diabetic nephropathy has been established in a new study [56]. Damage to mesangial cells caused by the primary pathogenic aspects of renal illness include an imbalance among the oxidation and antioxidant systems, as well as an excess of reactive oxygen species (ROS) [57]. Hyperglycemia has been shown to include glomerular mesangial cells and tubular epithelial cells to create excessive ROS, which damages tissue proteins, generates a significant number of lipid peroxides, and worsens renal oxidative damage [58]. Likewise, hyperglycemia has been shown to cause oxidative damage by causing upsurge in oxidative stress, which leads to an increase in ROS generation. Furthermore, due to the increased rate of oxygen consumption, renal tissues are more vulnerable to ROS and oxidative damage. Furthermore, an increase in MDA content, which is a strong sign of lipid peroxidation, might be caused by excessive ROS [59].

Oxidative stress is a key factor in the progression of hyperglycemia, and gingerols can play a very important role in fighting against oxidative stress [60,61]. It has been shown that 6-gingerol can regulate intracellular trafficking of glucose transporters in skeletal muscle cells, which is important for glucose metabolism [62]. The second phase of biphasic insulin release in response to glucose, which is necessary for glucose homeostasis, requires intracellular vesicular transport [63]. Therapy with 6-gingerol promotes glucose elimination in skeletal muscles by increasing glycogen synthase 1 activity and improving cell-surface presentation of GLUT4 transporters, according to Samad et al. [64]. As a result, 6-gingerol improves skeletal muscle glucose consumption by increasing GLUT4 membrane presentation [64]. In diabetic rats, Bhandari et al. found that an ethanolic extract of ginger administered orally for 20 days had substantial anti-hyperglycemic effects (*P* 0.01) [65]. In addition, Nammi et al. [66] reported that in an enriched fat diet, ginger extract in ethanol lowered body glucose levels, insulin levels total cholesterol, LDL, triglycerides, and phospholipids, and thus body weight.

## 5. Gingerol’s Restorative Role in Hyperglycemia Related Complications

### 5.1. Cardiomyopathy

**Figure 2:**
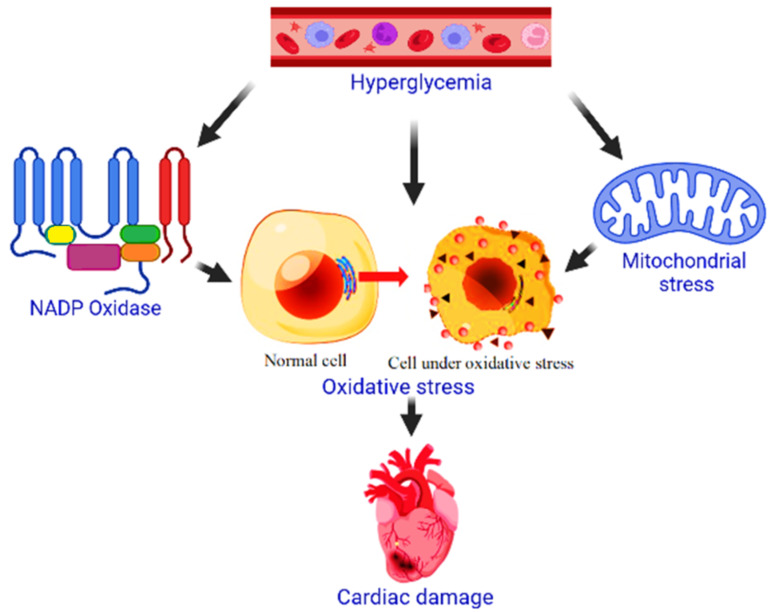
Role of NADPH oxidase and mitochondrial stress on cardiac injury due to hyperglycemia.

Abnormal regulation of lipid glycometabolism is one of the causes of hyperglycemia [67]. Diabetic cardiomyopathy (DCM) is a diabetic problem that develops as a result of changes in cardiac function and shape, as well as systemic hypertension, and is not caused by coronary artery disease. An increase in free fatty acids and blood lipoproteins can hasten the progression of cardiac and vascular complications [68,69,70,71]. DCM’s specific mechanism of action and its cause remain unknown. Excessive production of free radicals enhances the production of ROS and blocks the function of endogenous antioxidant defenses. Inflammatory reactions have a role in the progression of hyperglycemia or diabetes impairments; the inflammatory response accelerates the production of the delicate fat-cell response factor in hyperglycemic circumstances [72,73]. The evolution of diabetic complications is influenced by oxidative stress. The inflammatory response accelerates the formation of the delicate response factor of fat cells under hyperglycemic conditions, contributing to the progression of complications in diabetes [74,75]. It has also been reported that the main sources of stress in cardiomyocytes are the mitochondrial electron transport chain and the activities of the nicotinamide adenine dinucleotide phosphate (NADPH) oxidase. Myocardial damage is amplified by chronic hyperglycemia, which is intimately linked to this (Figure 2) [76].

Yu et al. [77] found that hyperglycemia produced an increase in glucose concentrations in the serum and plasma, due to the inhibition of insulin secretion caused by ROS. Increased ROS levels overwhelm the antioxidant defense mechanism, causing oxidative damage in pancreatic cells. In hyperglycemic rats, gingerol treatment resulted in a considerable reduction in serum and plasma glucose concentrations. This demonstrated lowered formation of free radicals and lipid peroxidation, while also protecting cells by increasing pancreatic cell insulin output and preventing oxidative stress.

By modulating inflammation, oxidative stress, metabolic anomalies, and cellular death pathways, Yu et al. [77] hypothesized that gingerol therapy had strong healing and therapeutic effectiveness against DCM and presumably other cardiovascular illnesses [78]. The effects of gingerol on oxidative stress and inflammatory variables verified the prevention of DCM in rats. The lowering of elevated triglycerides (TG) and blood glucose levels may be responsible for gingerol’s preventative impact. In another study M. El-Bassossy et al. [79] showed that oral treatment with 6-gingerol not only reduced hyperglycemia in diabetic rats, but also improved heart dysfunction caused by diabetes. Through a mechanism that is likely unrelated to its possible antioxidant impact, this ameliorative effect is obvious from improved contractility indices and ischemia, as shown in ECGs.

### 5.2. Nephropathy (Kidney Damage or Kidney Failure)

Diabetic nephropathy (DN) is among the very common microvascular concerns resulting from long-term hyperglycemia [54,80]. Persistent proteinuria, progressive loss of renal task, and structural alterations such as mesangial enlargement, glomerular hypertrophy, and intestinal fibrosis are all symptoms [54,55]. The significance of early blood glucose management in the anticipation of diabetic nephropathy has been verified experimentally [56]. Furthermore, the evolution of diabetic nephropathy is linked to hyperglycemic injury to mesangial cells [81,82]. The primary pathogenic causes in renal illness include an inequity between oxidant and antioxidant systems, as well as ROS production [57]. Chronic hyperglycemia causes oxidative stress, which is a significant factor in diabetic kidney disease progression. It causes cellular and metabolic problems such as protein oxidation, lipid peroxidation, and damage to DNA, which leads to cellular death [83]. Besides this, due to the increased rate of oxygen consumption, renal tissues are more vulnerable to ROS and oxidative damage. Furthermore, an upsurge in MDA concentration, which is a marker of the lipid peroxidation, might be caused by excessive ROS [59]. Hyperglycemia can lead to the production of excess ROS by glomerular mesangial cells and tubular epithelial cells. ROS can trigger a number of cellular responses that are important in the progression of hyperglycemia-induced kidney damage [84], associated with damaged tissue proteins, the creation of a significant quantity of lipid peroxides, and worsened oxidative damage to the kidneys [58,85,86,87,88].

Some issues, such as negative outcomes and inability to manage blood glucose levels, seriously restrict the therapeutic efficacy of such medications [89,90,91]. Moreover, the majority of oral hypoglycemic mediators used to treat diabetic nephropathy are adipogenic [92], and synthetic oral hypoglycemic medications have been associated with a slew of side effects [93]. As a result, there is a need to find medications that can protect against kidney damage while also limiting the negative consequences of diabetes [94]. It may be possible to treat diabetes using 6-gingerol. It exhibits strong insulin-secreting, lipid-lowering, anti-hyperglycemic, and antioxidant benefits in type II diabetic animal models [95].

According to Almatroodi et al. [18], treatment with 6-gingerol reduced renal dysfunction biomarkers such as serum creatinine and blood urea, indicating that 6-gingerol shields rental function in diabetic rats, and treatment with it significantly alleviated altered oxidative stress levels in STZ-induced diabetic animals, resulting in improved antioxidant levels. It has also been shown to lower MDA levels in the kidneys, whereas persistent hyperglycemia has been shown to cause a substantial rise in malondialdehyde in the kidneys of rats. In diabetic kidneys, extract of ginger effectively improves antioxidant levels [96]. Another study indicated that diabetic rats administered gentamicin in an enhanced gingerol solution had improved renal function measures, lower lipid peroxidation and nitrosative stress, and high GSH and SOD activity [21]. It has been shown that 6-gingerol alleviates gentamicin-induced renal cortex oxidative stress [97,98,99]. Antioxidant capacity and lipid peroxidation measures were used to investigate the effect of ginger powder on diabetic nephropathy. MDA levels were much lower in diabetic rats treated with ginger than in any other treatment group, according to Almatroodi et al., who found that ginger reduces lipid peroxidation, boosts antioxidant capacity, and reduces renal nephropathy [18,99,100].

Furthermore, it has been found that gingerol improves the state of renal tissue by altering p38MAPK and NF-κB activity, as well as controlling inflammatory reactions and oxidative stress [101]. It has been shown that 6-gingerol has renoprotective effects in diabetic rats due to its ability to regulate urea and creatinine levels, inhibiting oxidative stress, hyperglycemia, and inflammatory markers such as CRP, IL-6, IL-1, and TNF-α. Furthermore, in streptozotocin-induced diabetes, 6-gingerol has been shown to reduce kidney fibrosis and pathological alterations through lowering TNF-α-protein expression. To assess its hypoglycemic effects and particular dose in clinical practice, pharmacokinetic and bioavailability investigations are essential [18].

### 5.3. Retinopathy (Retinal Blood Vessel Damage)

Diabetic retinopathy (DR) is the most prevalent microvascular consequence of long-term hyperglycemia and the primary cause of vision impairment and blindness in adults all over the world. The overall prevalence of DR is 34.6% and vision-threatening DR has a prevalence of 10.2% [102]. A group of researchers extended these findings to worldwide numbers, predicting that the number of individuals living with DR will increase from 126.6 million in 2011 to 191.0 million by 2030 [103]. The microvascular alterations generated by hyperglycemia-induced metabolic and biochemical pathway activation are thought to be the cause of poor diabetic retinopathy outcomes. These pathways include increased synthesis of advanced glycation products (AGEs), activation of the polyol and hexosamine pathways, and activation of protein kinase C [104]. These processes, when combined, cause oxidative stress and inflammation, which compromises the integrity of vascular walls, resulting in blockage of vessels, higher permeability, and local ischemia [105,106].

The function of AGEs in diabetic vascular damage has been hypothesized. Inflammation, which is thought to be the major driver in the pathogenesis of DR, is exacerbated when AGEs are exposed to retinal vascular endothelial cells [107]. Improved manufacturing of pro-inflammatory cytokines, such as tumor necrosis factor alpha (TNF-α), has been linked to DR. TNF-α levels in vitro have been found to be higher in individuals with proliferative DR [108,109] and in the retinas of diabetic rats [108,110,111,112]. TNF-α stimulates leukocyte adherence to the endothelial cells of the retina and enhances the porousness of the blood–retinal barrier (BRB) [113]. The activation of nuclear factor kappa B (NF-kB) is linked to increased production of proinflammatory cytokines in DR [114]. NF-kB is the transcription factor that controls the generation of proinflammatory cytokines, which is important in the progression of DR [115,116]. The increased production of VEGF, which has emerged as a critical facilitator of BRB breakdown and neovascularization in DR, is linked to the exposure of vascular endothelial cells to AGEs [117]. VEGF is intrinsically connected to the development of DR and plays a crucial role in leukocyte-mediated BRB breakdown and retinal neovascularization [117]. Streptozotocin (STZ)-induced diabetic rat retinas have been shown to have increased VEGF expression [118,119,120], and VEGF, which is developed as a critical facilitator of breakdown and neovascularization of BRB in DR, is linked to the acquaintance of vascular endothelial cells to AGEs [121]. Current DR therapy options are ineffective, and disease progression frequently persists despite pharmaceutical and nonpharmacological therapies. Newer treatment strategies for DR that potentially target major mediators of microvascular damage are crucial [122]. It has also been shown that 6-gingerol has anti-angiogenesis properties in vivo and in vitro [123]. Dongare et al. explored whether 6-gingerol improves the microvascular alterations in DR. The thickness of the vascular basement membrane, the width of the retinal arteries, and hyperglycemia were all significantly reduced after taking ginger extract orally. When compared to the vehicle-treated group, the ginger extract-treated group showed a substantial improvement in the architecture of the retinal vasculature, as well as decreased expression of NFκ-B, TNF-α, and VEGF in the retinal tissue [124].

In the retinas of 6-gingerol-treated rats, Dongare et al. [122] found an improvement in the architecture of the retinal vasculature linked with considerably lower activity of TNF-κB and VEGF. Diabetic rats treated with 6-gingerol had a considerable drop in blood glucose levels and improved bodyweight preservation. The positive benefits of 6-gingerol in reducing hyperglycemia-induced structural abnormalities in retinal microvascular can be linked to its blood glucose-lowering effects. Ginger polyphenols have been shown to have hypoglycemic and insulinotropic effects [125,126]. In the absence of hyperglycemia, however, extract of ginger has been demonstrated to considerably lower the manufacturing of excess NF-κB and TNF-α [127,128]. In lipopolysaccharide-stimulated murine macrophages, ginger extract suppressed the PKC alpha and NF-κB pathways [128]. It has also been shown that gingerol prevents human endothelial cells from proliferating in response to VEGF [129,130]. These results suggest that gingerol’s effects on retinal vasculature are likely due in part to the compound’s direct anti-inflammatory and antiangiogenic properties, which are derived from its inhibition of NF-κB signaling, as well as TNF-α and VEGF activity. Although some of these effects might be attributed to ginger extract’s antihyperglycemic properties, it is more likely that they are attributable to the extract’s direct effects on the retinal vasculature. Even though the specific molecular targets are unclear at this time, gingerol appears to be a likely candidate for additional exploration [122].

By regulating NF-κB expression and lowering the expression of pro-inflammatory cytokines, gingerol can help to prevent retinal damage caused by hyperglycemia. The blocking of the AGE/RAGE/NF-κB pathway is thought to be the cause of zerumbone’s impact [131].

### 5.4. Cataract (Potentially Leading to Blindness, Clouding of the Normally Clear Lens of Your Eye)

Clouding of the ordinarily clear lens of the eye can lead to blindness [132]. AGE produced due to a hyperglycemic environment have been associated with a variety of pathologies, including diabetic cataracts. In rats, Saraswat et al. [133] found that eating ginger slowed not only the start but also the development of cataracts. Molecular analysis revealed that ginger consumption greatly reduced the production of numerous AGE products in the eye lens, including carboxymethyl lysine. In addition, ginger reduced osmotic stress in the lens caused by hyperglycemia. This suggests that ginger was helpful in preventing diabetic cataract formation in rats, owing to its antiglycating properties and, to a lesser extent, suppression of the polyol pathway.

### 5.5. Bone and Joint Problems

**Figure 3:**
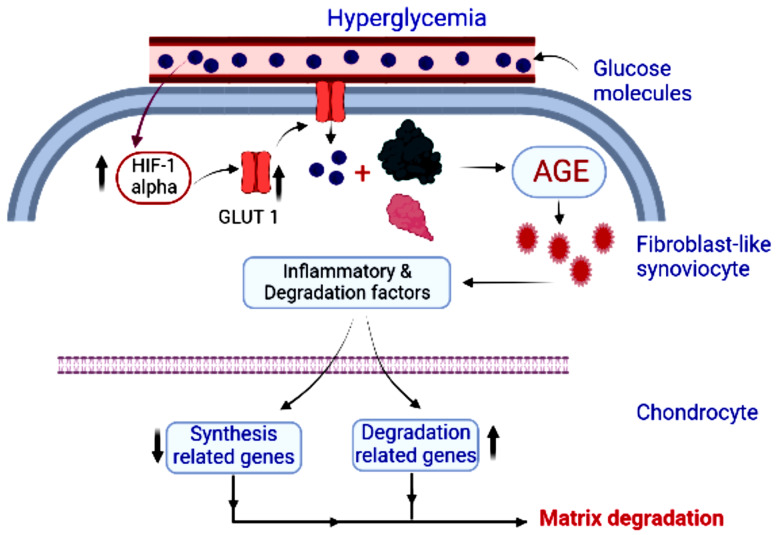
The activation of ERS and the release of proinflammatory substances from the synovium as a result of high glucose levels caused inflammation and degradation of the articular cartilage.

According to Li et al. [134] although osteoarthritis (OA) is strongly linked to diabetes, it is unknown how hyperglycemia causes or worsens OA. In cartilage metabolism and material exchange, synovium is crucial. Hyperglycemia causes AGE buildup in fibroblast-like synoviocytes (FLSs) via the HIF-1-GLUT1 pathway, which enhances the release of inflammatory agents from FLSs, causing chondrocyte breakdown and boosting the development of OA (Figure 3).

Ginger might help in the therapy of musculoskeletal problems [135]. Osteoarthritis [136,137] and hyperglycemia are examples of inflammation or inflammatory states [136]. Inhibition of prostaglandin and leukotriene construction is assumed to be the action mechanism [138].

One of the most important components of inflammation is 5-lipoxygenase, and lowering this factor helps to reduce inflammation. According to Flynn et al. [139] gingerol has considerable analgesic and anti-inflammatory properties through blocking PGE2 production. Young et al. [140] discovered that gingerol has analgesic and anti-inflammatory properties. For this investigation, female Wistar rats (180–240 g) and male ICR mice (18–22 g) were used. Doses of 50 mg/kg, 100 mg/kg, and 250 mg/kg of gingerol were given intraperitoneally. The findings demonstrated that paw edema was inhibited by gingerol at doses of 50 and 100 mg/kg.

The therapeutic efficacy of crude extract was compared to that of gingerol and its derivatives, a phytochemical ingredient. It was discovered that specific phytochemicals had a significant impact. Surprisingly, the crude extract that included essential oils and more polar chemicals had greater anti-inflammatory and anti-bone-degeneration properties. It was observed that non-gingerol substances, as well as gingerol, exhibited significant anti-arthritic efficacy. Another study, by Sharma et al., found that ginger oil had powerful anti-arthritic and anti-inflammatory properties [141]. Srivastava et al. 1992 discovered that ginger had anti-arthritic properties in people with rheumatoid arthritis, osteoarthritis, and muscle pain. [142]. Although most studies suggest that ginger’s anti-inflammatory properties operate via inhibiting COX-2 enzymes, Grzanna found that ginger inhibits both COX-1 and COX-2 enzymes. In addition to these findings, ginger was found to inhibit 5-lipoxygenase, which inhibits leukotriene production [143]. Nurtjahja-Tjendraputra et al. 2003 [144] also found that ginger inhibits COX-1 activity, showing that ginger’s 8-paradol is a powerful COX-1 inhibitor.

Lantz et al. also found gingerols to have potent anti-inflammatory effects, but they focused on its ability to suppress LPS-induced COX2 expression [42]. Pragasam et al. conducted both in vivo and in vitro tests to assess the effect of gingerol as an inflammatory agent. In 2011, a mouse model of gouty arthritis was established using monosodium urate crystal-induced inflammation. They discovered that gingerol inhibited lactate dehydrogenase and acid phosphate, as well as reducing the quantity of lysosomal enzymes, based on which they concluded that ginger phytochemicals have anti-inflammatory properties [145].

Further research has been done on how to reduce chronic inflammation or arthritis swelling before it leads to extensive and irreparable bone loss. Lee et al. [146] found that gingerol suppressed NF- nuclear activation and protein kinase C (PKC) translocation in lipopolysaccharide stimulated macrophages, preventing Ca^+2^ influx and altering mitochondrial membrane potential. As a result, inducible nitric oxide synthase and TNF-α expression were lowered and inflammation was reduced. Moreover, 1-dehydro-10-gingerdione has been identified an important anti-inflammatory, reducing the NF-kB-controlled expression of inflammatory genes linked to innate immunity through toll-like receptors (TLRs) [147]. In this study, the researchers employed RAW 264.7 macrophages or primary macrophages extracted from the bone marrow of C57BL/6 or C3H/HeJ mice which had been stimulated with the TLR agonist LPS in the presence of 1-dehydro-10-gingerdione. A kinase assay and immunoblot analysis were used to determine the catalytic activity of inhibitory 𝜅B (I𝜅B) kinase b (IKK𝛽). TLR4 agonists or TNF-α-induced cytoplasmic IKK𝛽-catalyzed I𝜅B phosphorylation was demonstrated to be blocked irreversibly by 1-dehydro-10-gingerdione in macrophages. When the Cys179 in IKK𝛽’s activation loop was replaced with Ala, the effects of 1-dehydro-10-gingerdione were reversed, indicating that the compound possessed a direct contact site. Finally, in LPS-stimulated macrophages, 1-dehydro-10-gingerdione inhibited NF-𝜅B-regulated gene production of inducible NOS, COX2, and IL-6, suggesting that NF-𝜅B activation was disrupted [148].

### 5.6. Periodontitis (Tooth and Gum Infection)

Periodontitis is a condition that develops as a result of localized infections in the oral cavity that permanently destroy the mechanism of attachment of the tooth via the root cementum, peripheral ligament, and alveolar bone. Over time, the delicate gums and bone that surrounds the teeth disintegrate. This may result in tooth loss [149].

Hyperglycemia appears to be a risk factor for greater periodontal damage, although treating periodontitis can also help with glycemic management. Hyperglycemia and periodontitis have a bidirectional relationship, according to epidemiological data. Hyperglycemia can affect microbial assemblages in the subgingival space, disrupt functions inside the cell, and modify the metabolism of collagen. The production of AGEs can alter the extracellular matrix further and receptor binding in cells can exacerbate inflammation. Further, periodontitis causes hyperlipidemia and insulin resistance [150].

Dental researchers are fully aware of the high frequency of periodontitis in people with hyperglycemia. Periodontal disease has been associated to the generation of proinflammatory mediators connected to hyperglycemia (IL-6, TNF-α, and CRP) [151].

Gingerol-related components have been shown to prevent the proliferation of germs in the mouth linked with chronic peritonitis in the human oral cavity [152]. Javid et al. [153] published a paper with similar findings. Menon et al. [110] found that ginger powder is efficient for treating irritation of the gums and painful discomfort following an open flap debridement. This is because it has anti-inflammatory and analgesic characteristics, making it a good alternative to synthetic drugs such as Ibuprofen [154].

## 6. Limitations

Ginger’s medicinal potential as an antioxidant with anti-inflammatory, anti-hyperglycemic, and anti-cancer properties has been extensively researched and published. Almost all published studies concern whole ginger extract. However, there are few investigations into the function of gingerol in hyperglycemia. Furthermore, research shows that 6-gingerol is the form of gingerol most commonly utilized to treat hyperglycemia and associated disorders. Other variants of gingerol, such as 4-gingerol, 8-gingerol, 10-gingerol, and 12-gingerol, should be investigated for their potential medicinal use in the treatment of hyperglycemia [10]. More research into all forms of gingerols is needed in order to establish it as an anti-hyperglycemic medicine and to treat different hyperglycemia-related disorders. In comparison to 6-gingerol, 4-, 8-, 10-, and 12-gingerol may be more effective in the treatment of hyperglycemia and associated illnesses. The effects of different concentrations of these gingerols must also be investigated in order to determine the appropriate dosage for treating hyperglycemia and related disorders. Furthermore, there has been no study on the use of gingerol to treat hyperglycemia-induced neuropathy; however, one study was conducted in this context on the use of whole ginger extract to reduce peripheral neuropathy in diabetic rats provoked by streptozotocin [155]. Farjrin et al. [156] conducted research on mice, employing 6-shogaol to alleviate severe diabetic neuropathy. As a result, more studies into gingerol’s function in hyperglycemia-induced neuropathy are required.

## 7. Conclusions

Gingerol is chemically linked to capsaicin, the fiery component found in chili peppers, and piperine, a substance found in black pepper. Its natural antioxidant properties are behind its extreme potency in preventing and curing a variety of ailments. It is a possible medication for hyperglycemia and its resultant potentially morbidity-causing disorders, such as cardiomyopathy, nephropathy, retinopathy, cataract, bone and joint problems, and periodontitis. It does so by modulating inflammation, oxidative stress and metabolic anomalies. It has also been shown to prevent the growth of oral bacteria connected to chronic peritonitis in the oral cavity. Studies have been conducted in this area, although the majority are focused on 6-gingerol, the most prevalent component of ginger, which shows considerable promise in the treatment of hyperglycemia and the diseases that accompany it. Clinical trials are, however, needed to prove gingerol’s medicinal effectiveness.
